# Graded Modal Dependent Type Theory

**DOI:** 10.1007/978-3-030-72019-3_17

**Published:** 2021-03-23

**Authors:** Benjamin Moon, Harley Eades III, Dominic Orchard

**Affiliations:** Imperial College, London, UK; 9 University of Kent, Canterbury, UK; 10 Augusta University, Augusta, USA

## Abstract

Graded type theories are an emerging paradigm for augmenting the reasoning power of types with parameterizable, fine-grained analyses of program properties. There have been many such theories in recent years which equip a type theory with quantitative dataflow tracking, usually via a semiring-like structure which provides analysis on variables (often called ‘quantitative’ or ‘coeffect’ theories). We present Graded Modal Dependent Type Theory (Grtt for short), which equips a dependent type theory with a general, parameterizable analysis of the flow of data, both in and between computational terms and types. In this theory, it is possible to study, restrict, and reason about data use in programs and types, enabling, for example, parametric quantifiers and linearity to be captured in a dependent setting. We propose Grtt, study its metatheory, and explore various case studies of its use in reasoning about programs and studying other type theories. We have implemented the theory and highlight the interesting details, including showing an application of grading to optimising the type checking procedure itself.

## 1 Introduction

The difference between simply-typed, polymorphically-typed, and dependently-typed languages can be characterised by the *dataflow* permitted by each type theory. In each, dataflow can be enacted by *substituting* a term for occurrences of a variable in another term, the scope of which is delineated by a binder. In the simply-typed $\lambda$-calculus, data can only flow in 'computational' terms; computations and types are separate syntactic categories, with variables, bindings ($\lambda$), and substitution—and thus dataflow—only at the computational level. In contrast, polymorphic calculi like System F [26,52] permit dataflow within types, via type quantification ($\forall$), and a limited form of dataflow from computations to types, via type abstraction ($\Lambda$) and type application. Dependently-typed calculi (e.g., [14,40,41,42]) break down the barrier between computations and types further: variables are bound simultaneously in types and computations, such that data can flow both to computations and types via dependent functions ($\Pi$) and application. This pervasive dataflow enables the Curry-Howard correspondence to be leveraged for program reasoning and theorem proving [59]. However, unrestricted dataflow between computations and types can impede reasoning and can interact poorly with other type theoretic ideas.

Firstly, System F allows *parametric reasoning* and notions of representation independence [53,57], but this is lost in general in dependently-typed languages when quantifying over higher-kinded types [45] (rather than just 'small' types [7,36]). Furthermore, unrestricted dataflow impedes efficient compilation as compilers do not know, from the types alone, where a term is actually needed. Additional static analyses are needed to recover dataflow information for optimisation and reasoning. For example, a term shown to be used only for type checking (not flowing to the computational 'run time' level) can be erased [9]. Thus, dependent theories do not expose the distinction between proof relevant and irrelevant terms, requiring extensions to capture irrelevance [4,50,51]. Whilst unrestricted dataflow between computations and terms has its benefits, the permissive nature of dependent types can hide useful information. This permissiveness also interacts poorly with other type theories which seek to deliberately restrict dataflow, notably *linear types*.

Linear types allow data to be treated as a 'resource' which must be consumed exactly once: linearly-typed values are restricted to linear dataflow [27,58,60]. Reasoning about resourceful data has been exploited by several languages, e.g., ATS [54], Alms [56], Clean [18], Granule [46], and Linear Haskell [8]. However, linear dataflow is rare in a dependently-typed setting. Consider typing the body of the polymorphic identity function in Martin-Löf type theory:

$$a : \mathsf{Type}, x : a \vdash x : a$$

This judgment uses $a$ twice (typing $x$ in the context and the subject of the judgment) and $x$ once in the term but not at all in the type. There have been various attempts to meaningfully reconcile linear and dependent types [12,15,37,39] usually by keeping them separate, allowing types to depend only on non-linear variables. All such theories cannot distinguish variables used for computation from those used purely for type formation, which could be erased at runtime.

Recent work by McBride [43], refined by Atkey [6], generalises ideas from 'coeffect analyses' (variable usage analyses, like that of Petricek et al. [49]) to a dependently-typed setting to reconcile the ubiquitous flow of data in dependent types with the restricted dataflow of linearity. This approach, called Quantitative Type Theory (QTT), types the above example as:

$$a \overset{0}{:} \mathsf{Type}, x \overset{1}{:} a \vdash x \overset{1}{:} a$$

The annotation 0 on $a$ explains that we can use $a$ to form a type, but we cannot, or do not, use it at the term level, thus it can be erased at runtime. The cornerstone of QTT's approach is that dataflow of a term to the type level counts as 0 use, so arbitrary type-level use is allowed whilst still permitting quantitative analysis of computation-level dataflow. Whilst this gives a useful way to relate linear and dependent types, it cannot however reason about dataflow at the type-level (all type-level usage counts as 0). Thus, for example, QTT cannot express that a variable is used just computationally but not at all in types.

In an extended abstract, Abel proposes a generalisation of QTT to track variable use in both types and computations [2], suggesting that tracking in types enables type checking optimisations and increased expressivity. We develop a core dependent type theory along the same lines, using the paradigm of *grading*: graded systems augment types with additional information, capturing the structure of programs [23,46]. We therefore name our approach *Graded Modal Dependent Type Theory* (GRTT for short). Our type theory is parameterised by a semiring which, like other coeffect and quantitative approaches [3,6,10,25,43,49,61], describes dataflow through a program, but in *both types and computations equally*, remedying QTT's inability to track type-level use. We extend Abel's initial idea by presenting a rich language, including dependent tensors, a complete metatheory, and a *graded modality* which aids the practical use of this approach (e.g., enabling functions to use components of data non-uniformly). The result is a calculus which extends the power of existing non-dependent graded languages, like Granule [46], to a dependent setting.

We begin with the definition of GRTT in Section 2, before demonstrating the power of GRTT through case studies in Section 3, where we show how to use grading to restrict GRTT terms to simply-typed reasoning, parametric reasoning (regaining universal quantification smoothly within a dependent theory), existential types, and linear types. The calculus can be instantiated to different kinds of dataflow reasoning: we show an example application to information-flow security. We then show the metatheory of GRTT in Section 4: admissibility of graded structural rules, substitution, type preservation, and strong normalisation.

We implemented a prototype language based on GRTT called **Gerty**.[3] We briefly mention its syntax in Section 2.5 for use in examples. Later, Section 5 describes how the formal definition of GRTT is implemented as a bidirectional type checking algorithm, interfacing with an SMT solver to solve constraints over grades. Furthermore, Abel conjectured that a quantitative dependent theory could enable usage-based optimisation of type-checking itself [2], which would assist dependently-typed programming at scale. We validate this claim in Section 5 showing a grade-directed optimisation to **Gerty**'s type checker.

Section 6 discusses next steps for increasing the expressive power of GRTT. Full proofs and details are provided in the extended version of this paper [44].

**Gerty** has some similarity to Granule [46]: both are functional languages with graded types. However, Granule has a linearly typed core and no dependent types (only indexed types), thus has no need for resource tracking at the type level (type indices are not subject to tracking and their syntax is restricted).

## 2 GrTT: Graded Modal Dependent Type Theory

GRTT augments a standard presentation of dependent type theory with 'grades' (elements of a semiring) which account for how variables are used, i.e., their *dataflow*. Whilst existing work uses grades to describe usage only in computational terms (e.g. [10]), GRTT incorporates additional grades to account for how variables are used in types. We introduce here the syntax and typing, and briefly show the syntax of the implementation. Section 4 describes its metatheory.

[3] https://github.com/granule-project/gerty/releases/tag/esop2021

### 2.1 Syntax

The syntax of GRTT is that of a standard Martin-Löf type theory, with the addition of a *graded modality* and grade annotations on function and tensor binders. Throughout, $s$ and $r$ range over grades, which are elements of a semiring $(\mathcal{R}, *, 1, +, 0)$. It is instructive to instantiate this semiring to the natural number semiring $(\mathbb{N}, \times, 1, +, 0)$, which captures the exact number of times variables are used. We appeal to this example in descriptions here.

GRTT has a single syntactic sort for computations and types:

$$
\begin{array}{llll}
(\textit{terms}) & t, A, B, C ::= x & \mid \mathsf{Type}_l & \\
& \mid (x :_{(s,r)} A) \to B & \mid \lambda x.t & \mid t_1\, t_2 \\
& \mid (x :_r A) \otimes B & \mid (t_1, t_2) & \mid \mathsf{let}\,(x, y) = t_1 \,\mathsf{in}\, t_2 \\
& \mid \Box_s A & \mid \Box t & \mid \mathsf{let}\, \Box x = t_1 \,\mathsf{in}\, t_2 \\
(\textit{levels}) & l ::= 0 \mid \mathsf{suc}\ l \mid l_1 \sqcup l_2 & &
\end{array}
$$

Terms include variables and a constructor for an inductive hierarchy of universes, annotated by a level $l$. Dependent function types are annotated with a pair of grades $s$ and $r$, with $s$ capturing how $x$ is used in the body of the inhabiting function and $r$ capturing how $x$ is used in the codomain $B$. Dependent tensors have a single grade $r$, which describes how the first element is used in the typing of the second. The graded modal type operator $\Box_s A$ 'packages' a term and its dependencies so that values of type $A$ can be used with grade $s$ in the future. Graded modal types are introduced via *promotion* $\Box t$ and eliminated via $\mathsf{let}\, \Box x = t_1 \,\mathsf{in}\, t_2$. The following sections explain the semantics of each piece of syntax with respect to its typing. We typically use $A$ and $B$ to connote terms used as types.

### 2.2 Typing Judgments, Contexts, and Grading

Typing judgments are written in either of the following two equivalent forms:

$$
(\Delta \mid \sigma_1 \mid \sigma_2) \odot \Gamma \vdash t : A \qquad\qquad \begin{pmatrix} \Delta \\ \sigma_1 \\ \sigma_2 \end{pmatrix} \odot \Gamma \vdash t : A
$$

The 'horizontal' syntax (left) is used most often, with the equivalent 'vertical' form (right) used for clarity in some places. Ignoring the part to the left of $\odot$, typing judgments and their rules are essentially those of Martin-Löf type theory (with the addition of the modality) where $\Gamma$ ranges over usual dependently-typed typing *contexts*. The left of $\odot$ provides the grading information, where $\sigma$ and $\Delta$ range over *grade vectors* and *context grade vectors* respectively, of the form:

$$
\begin{array}{ccc}
(\textit{contexts}) & (\textit{grade vectors}) & (\textit{context grade vectors}) \\
\Gamma ::= \emptyset \mid \Gamma, x : A & \sigma ::= \emptyset \mid \sigma, s & \Delta ::= \emptyset \mid \Delta, \sigma
\end{array}
$$

A grade vector $\sigma$ is a vector of semiring elements, and a context vector $\Delta$ is a vector of grade vectors. We write $(s_1, \ldots, s_n)$ to denote an $n$-vector and likewise for context grade vectors. We omit parentheses when this would not cause ambiguity. Throughout, a comma is used to concatenate vectors and disjoint contexts, and to extend vectors with a single grade, grade vector, or typing assumption.

For a judgment $(\Delta \mid \sigma_s \mid \sigma_r) \odot \Gamma \vdash t : A$ the vectors $\Gamma$, $\Delta$, $\sigma_s$, and $\sigma_r$ are all of equal size. Given a typing assumption $y : B$ at index $i$ in $\Gamma$, the grade $\sigma_s[i] \in \mathcal{R}$ denotes the use of $y$ in $t$ (the *subject* of the judgment), the grade $\sigma_r[i] \in \mathcal{R}$ denotes the use of $y$ in $A$ (the *subject's type*), and $\Delta[i] \in \mathcal{R}^i$ (of size $i$) describes how assumptions prior to $y$ are used to form $y$'s type, $B$.

Consider the following example, which types the body of a function that takes two arguments of type $a$, and returns only the first:

$$\begin{pmatrix} (),(1),(1,0) \\ 0,1,0 \\ 1,0,0 \end{pmatrix} \odot a : \mathsf{Type}_l, x : a, y : a \vdash x : a$$

Let the context grade vector be called $\Delta$. Then, $\Delta[0] = ()$ (empty vector) explains that there are no assumptions that are used to type $a$ in the context, as $\mathsf{Type}_l$ is a closed term and the first assumption. $\Delta[1] = (1)$ explains that the first assumption $a$ is used (grade 1) in the typing of $x$ in the context, and $\Delta[2] = (1, 0)$, explains that $a$ is used once in the typing of $y$ in the context, and $x$ is unused in the typing of $y$. The subject grade vector $\sigma_s = (0, 1, 0)$ explains that $a$ is unused in the subject, $x$ is used once, and $y$ is unused. Finally, the subject type vector $\sigma_r = (1, 0, 0)$ explains that $a$ appears once in the subject's type (which is just $a$), and $x$ and $y$ are unused in the formation of the subject's type.

To aid reading, recall that standard typing rules typically have the form *context* $\vdash$ *subject* : *subject-type*, the order of which is reflected by $(\Delta \mid \sigma_s \mid \sigma_r) \odot \ldots$ giving the context, subject, and subject-type grading respectively.

*Well-formed Contexts* The relation $\Delta \odot \Gamma \vdash$ identifies a context $\Gamma$ as well-formed with respect to context grade vector $\Delta$, defined by the following rules:

$$\frac{}{\emptyset \odot \emptyset \vdash}\ \text{WF}\emptyset \qquad \frac{(\Delta \mid \sigma \mid \mathbf{0}) \odot \Gamma \vdash A : \mathsf{Type}_l}{\Delta, \sigma \odot \Gamma, x : A \vdash}\ \text{WFEXT}$$

Unlike typing, well-formedness does not need to include subject and subject-type grade vectors, as it considers only the well-formedness of the assumptions in a context with respect to prior assumptions in the context. The WF$\emptyset$ rule states that the empty context is well-formed with an empty context grade vector as there are no assumptions to account for. The WFEXT rule states that given $A$ is a type under the assumptions in $\Gamma$, with $\sigma$ accounting for the usage of $\Gamma$ variables in $A$, and $\Delta$ accounting for usage within $\Gamma$, then we can form the well-formed context $\Gamma, x : A$ by extending $\Delta$ with $\sigma$ to account for the usage of $A$ in forming the context. The notation $\mathbf{0}$ denotes a vector for which each element is the semiring 0. Note that the well-formedness $\Delta \odot \Gamma \vdash$ is inherent from the premise of WFEXT due to the following lemma:

**Lemma 1 (Typing contexts are well-formed).** *If* $(\Delta \mid \sigma_1 \mid \sigma_2) \odot \Gamma \vdash t : A$ *then* $\Delta \odot \Gamma \vdash$.

## 2.3 Typing Rules

We examine the typing rules of GRTT one at a time.

Variables are introduced as follows:

$$\frac{(\Delta_1, \sigma, \Delta_2) \odot \Gamma_1, x : A, \Gamma_2 \vdash \quad |\Delta_1| = |\Gamma_1|}{(\Delta_1, \sigma, \Delta_2 \mid \mathbf{0}^{|\Delta_1|}, 1, \mathbf{0} \mid \sigma, 0, \mathbf{0}) \odot \Gamma_1, x : A, \Gamma_2 \vdash x : A}\ \textsc{Var}$$

The premise identifies $\Gamma_1, x : A, \Gamma_2$ as well-formed under the context grade vector $\Delta_1, \sigma, \Delta_2$. By the size condition $|\Delta_1| = |\Gamma_1|$, we are able to identify $\sigma$ as capturing the usage of the variables $\Gamma_1$ in forming $A$. This information is used in the conclusion, capturing type-level variable usage as $\sigma, 0, \mathbf{0}$, which describes that $\Gamma_1$ is used according to $\sigma$ in the subject's type ($A$), and that the $x$ and the variables of $\Gamma_2$ are used with grade 0. For subject usage, we annotate the first zero vector with a size $|\Delta_1|$, allowing us to single out $x$ as being the only assumption used with grade 1 in the subject; all other assumptions are used with grade 0.

For example, typing the body of the polymorphic identity ends with Var:

$$\frac{\dfrac{\cdots}{((), (1)) \odot a : \mathsf{Type}, x : a \vdash}\ \textsc{wfExt} \qquad |(())| = |a : \mathsf{Type}|}{(((), (1)) \mid 0, 1 \mid 1, 0) \odot a : \mathsf{Type}, x : a \vdash x : a}\ \textsc{Var}$$

The premise implies that $((), 1, 0) \odot a : \mathsf{Type} \vdash a : \mathsf{Type}$ by the following lemma:

**Lemma 2 (Typing an assumption in a well-formed context).** *If* $\Delta_1, \sigma, \Delta_2 \odot \Gamma_1, x : A, \Gamma_2 \vdash$ *with* $|\Delta_1| = |\Gamma_1|$*, then* $(\Delta_1 \mid \sigma \mid \mathbf{0}) \odot \Gamma_1 \vdash A : \mathsf{Type}_l$ *for some* $l$*.*

In the conclusion of Var, the typing $((), 1, 0) \odot a : \mathsf{Type} \vdash a : \mathsf{Type}$ is 'distributed' to the typing of $x$ in the context and to the formation the subject's type. Thus subject grade $(0, 1)$ corresponds to the absence of $a$ from the subject and the presence of $x$, and subject-type grade $(1, 0)$ corresponds to the presence of $a$ in the subject's type ($a$), and the absence of $x$.

Typing universes are formed as follows:

$$\frac{\Delta \odot \Gamma \vdash}{(\Delta \mid \mathbf{0} \mid \mathbf{0}) \odot \Gamma \vdash \mathsf{Type}_l : \mathsf{Type}_{\mathsf{suc}\ l}}\ \mathsf{Type}$$

We use an inductive hierarchy of universes [47] with ordering $<$ such that $l < \mathsf{suc}\ l$. Universes can be formed under any well-formed context, with every assumption graded with 0 subject and subject-type use, capturing the absence of any assumptions from the universes, which are closed forms.

*Functions* Function types $(x :_{(s,r)} A) \to B$ are annotated with two grades: explaining that $x$ is used with grade $s$ in the body of the inhabiting function and with grade $r$ in $B$. Function types have the following formation rule:

$$\frac{(\Delta \mid \sigma_1 \mid \mathbf{0}) \odot \Gamma \vdash A : \mathsf{Type}_{l_1} \quad (\Delta, \sigma_1 \mid \sigma_2, r \mid \mathbf{0}) \odot \Gamma, x : A \vdash B : \mathsf{Type}_{l_2}}{(\Delta \mid \sigma_1 + \sigma_2 \mid \mathbf{0}) \odot \Gamma \vdash (x :_{(s,r)} A) \to B : \mathsf{Type}_{l_1 \sqcup l_2}} \to$$

The usage of the dependencies of $A$ and $B$ (excepting $x$) are given by $\sigma_1$ and $\sigma_2$ in the premises (in the 'subject' position) which are combined as $\sigma_1 + \sigma_2$ (via pointwise vector addition using the + of the semiring), which serves to *contract* the dependencies of the two types. The usage of $x$ in $B$ is captured by $r$, and then internalised to the binder in the conclusion of the rule. An arbitrary grade for $s$ is allowed here as there is no information on how $x$ is used in an inhabiting function body. Function terms are then typed by the following rule:

$$\frac{(\Delta, \sigma_1 \mid \sigma_3, r \mid \mathbf{0}) \odot \Gamma, x : A \vdash B : \mathsf{Type}_l \quad (\Delta, \sigma_1 \mid \sigma_2, s \mid \sigma_3, r) \odot \Gamma, x : A \vdash t : B}{(\Delta \mid \sigma_2 \mid \sigma_1 + \sigma_3) \odot \Gamma \vdash \lambda x.t : (x :_{(s,r)} A) \to B} \lambda_i$$

The second premise types the body of the λ-term, showing that $s$ captures the usage of $x$ in $t$ and $r$ captures the usage of $x$ in $B$; the subject and subject-type grades of $x$ are then internalised as annotations on the function type's binder.

Dependent functions are eliminated through application:

$$\frac{\begin{array}{l}(\Delta, \sigma_1 \mid \sigma_3, r \mid \mathbf{0}) \odot \Gamma, x : A \vdash B : \mathsf{Type}_l \\ (\Delta \mid \sigma_2 \mid \sigma_1 + \sigma_3) \odot \Gamma \vdash t_1 : (x :_{(s,r)} A) \to B \quad (\Delta \mid \sigma_4 \mid \sigma_1) \odot \Gamma \vdash t_2 : A\end{array}}{(\Delta \mid \sigma_2 + s * \sigma_4 \mid \sigma_3 + r * \sigma_4) \odot \Gamma \vdash t_1\, t_2 : [t_2/x]B} \lambda_e$$

where $*$ is the scalar multiplication of a vector, using the semiring multiplication. Given a function $t_1$ which uses its parameter with grade $s$ to compute and with grade $r$ in the typing of the result, we can apply it to a term $t_2$, provided that we have the resources required to form $t_2$ scaled by $s$ at the subject level and by $r$ at the subject-type level, since $t_2$ is substituted into the return type $B$. This scaling behaviour is akin to that used in coeffect calculi [25,49], QTT [6,43] and Linear Haskell [8], but scalar multiplication happens here at both the subject and subject-type level. The use of variables in $A$ is accounted for by $\sigma_1$ as explained in the third premise, but these usages are not present in the resulting application since $A$ no longer appears in the types or the terms.

Consider the constant function $\lambda x.\lambda y.x : (x :_{(1,0)} A) \to (y :_{(0,0)} B) \to A$ (for some $A$ and $B$). Here the resources required for the second parameter will always be scaled by 0, which is absorbing, meaning that anything passed as the second argument has 0 subject and subject-type use. This example begins to show some of the power of grading—the grades capture the program structure at all levels.

*Tensors* The rule for forming dependent tensor types is as follows:

$$\frac{(\Delta \mid \sigma_1 \mid \mathbf{0}) \odot \Gamma \vdash A : \mathsf{Type}_l \quad (\Delta, \sigma_1 \mid \sigma_2, r \mid \mathbf{0}) \odot \Gamma, x : A \vdash B : \mathsf{Type}_l}{(\Delta \mid \sigma_1 + \sigma_2 \mid \mathbf{0}) \odot \Gamma \vdash (x :_r A) \otimes B : \mathsf{Type}_l} \otimes$$

This rule is almost identical to function type formation $\to$ but with only a single grade $r$ on the binder, since $x$ is only bound in $B$ (the type of the second component), and not computationally. For 'quantitative' semirings, where 0 really means unused (see Section 3), $(x :_0 A) \otimes B$ is then a product $A \times B$.

Dependent tensors are introduced as follows:

$$\frac{\begin{array}{l}(\Delta, \sigma_1 \mid \sigma_3, r \mid \mathbf{0}) \odot \Gamma, x : A \vdash B : \mathsf{Type}_l \\ (\Delta \mid \sigma_2 \mid \sigma_1) \odot \Gamma \vdash t_1 : A \qquad (\Delta \mid \sigma_4 \mid \sigma_3 + r * \sigma_2) \odot \Gamma \vdash t_2 : [t_1/x]B\end{array}}{(\Delta \mid \sigma_2 + \sigma_4 \mid \sigma_1 + \sigma_3) \odot \Gamma \vdash (t_1, t_2) : (x :_r A) \otimes B} \otimes_i$$

In the typing premise for $t_2$, occurrences of $x$ are replaced with $t_1$ in the type, ensuring that the type of the second component ($t_2$) is calculated using the first component ($t_1$). The resources for $t_1$ in this substitution are scaled by $r$, accounting for the existing usage of $x$ in $B$. In the conclusion, we see the resources for the two components (and their types) combined via the semiring addition.

Finally, tensors are eliminated with the following rule:

$$\frac{\begin{array}{l}(\Delta \mid \sigma_3 \mid \sigma_1 + \sigma_2) \odot \Gamma \vdash t_1 : (x :_r A) \otimes B \\ (\Delta, (\sigma_1 + \sigma_2) \mid \sigma_5, r' \mid \mathbf{0}) \odot \Gamma, z : (x :_r A) \otimes B \vdash C : \mathsf{Type}_l \\ (\Delta, \sigma_1, (\sigma_2, r) \mid \sigma_4, s, s \mid \sigma_5, r', r') \odot \Gamma, x : A, y : B \vdash t_2 : [(x, y)/z]C\end{array}}{(\Delta \mid \sigma_4 + s * \sigma_3 \mid \sigma_5 + r' * \sigma_3) \odot \Gamma \vdash \mathsf{let}\,(x, y) = t_1 \,\mathsf{in}\, t_2 : [t_1/z]C} \otimes_e$$

As this is a dependent eliminator, we allow the result type $C$ to depend upon the value of the tensor as a whole, bound as $z$ in the second premise with grade $r'$, into which is substituted our actual tensor term $t_1$ in the conclusion.

Eliminating a tensor ($t_1$) requires that we consider each component ($x$ and $y$) is used with the same grade $s$ in the resulting expression $t_2$, and that we scale the resources of $t_1$ by $s$. This is because we cannot inspect $t_1$ itself, and semiring addition is not injective (preventing us from splitting the grades required to form $t_1$). This prevents forming certain functions (e.g., projections) under some semirings, but this can be overcome by the introduction of *graded modalities*.

*Graded Modality* Graded binders alone do not allow different parts of a value to be used differently, e.g., computing the length of a list ignores the elements, projecting from a pair discards one component. We therefore introduce a *graded modality* (à la [10,46]) allowing us to capture the notion of local inspection on data and internalising usage information into types. A type $\Box_s A$ denotes terms of type $A$ that are used with grade $s$. Type formation and introduction rules are:

$$\frac{(\Delta \mid \sigma \mid \mathbf{0}) \odot \Gamma \vdash A : \mathsf{Type}_l}{(\Delta \mid \sigma \mid \mathbf{0}) \odot \Gamma \vdash \Box_s A : \mathsf{Type}_l} \Box \qquad \frac{(\Delta \mid \sigma_1 \mid \sigma_2) \odot \Gamma \vdash t : A}{(\Delta \mid s * \sigma_1 \mid \sigma_2) \odot \Gamma \vdash \Box t : \Box_s A} \Box_i$$

To form a term of type $\Box_s A$, we 'promote' a term $t$ of type $A$ by requiring that we can use the resources used to form $t$ ($\sigma_1$) according to grade $s$. This 'promotion' resembles that of other graded modal systems (e.g., [3,10,23,46]), but the elimination needs to also account for type usage due to dependent elimination.

We can see promotion $\Box_i$ as capturing $t$ for later use according to grade $s$. Thus, when eliminating a term of type $\Box_s A$, we must consider how the 'unboxed' term is used with grade $s$, as per the following dependent eliminator:

$$\frac{\begin{array}{l}(\Delta, \sigma_2 \mid \sigma_4, r \mid \mathbf{0}) \odot \Gamma, z : \Box_s A \vdash B : \mathsf{Type}_l \\ (\Delta \mid \sigma_1 \mid \sigma_2) \odot \Gamma \vdash t_1 : \Box_s A \quad (\Delta, \sigma_2 \mid \sigma_3, s \mid \sigma_4, (s * r)) \odot \Gamma, x : A \vdash t_2 : [\Box x/z]B\end{array}}{(\Delta \mid \sigma_1 + \sigma_3 \mid \sigma_4 + r * \sigma_1) \odot \Gamma \vdash \mathsf{let}\,\Box x = t_1 \,\mathsf{in}\, t_2 : [t_1/z]B} \Box_e$$

This rule can be understood as a kind of 'cut', connecting a 'capability' to use a term of type $A$ according to grade $s$ with the requirement that $x : A$ is used according to grade $s$ as a dependency of $t_2$. Since we are in a dependently-typed setting, we also substitute $t_1$ into the type level such that $B$ can depend on $t_1$ according to grade $r$ which then causes the dependencies of $t_1$ ($\sigma_1$) to be scaled-up by $r$ and added to the subject-type grading.

*Equality, Conversion, and Subtyping* A key part of dependent type theories is a notion of term equality and type conversion [33]. GRTT term equality is via judgments $(\Delta \mid \sigma_1 \mid \sigma_2) \odot \Gamma \vdash t_1 = t_2 : A$ equating terms $t_1$ and $t_2$ of type $A$. Equality includes full congruences as well as $\beta\eta$-equality for functions, tensors, and graded modalities, of which the latter are:

$$\frac{\begin{array}{c}(\Delta, \sigma_2 \mid \sigma_4, r \mid \mathbf{0}) \odot \Gamma, z : \Box_s A \vdash B : \mathsf{Type}_l \\ (\Delta \mid \sigma_1 \mid \sigma_2) \odot \Gamma \vdash t_1 : A \quad (\Delta, \sigma_2 \mid \sigma_3, s \mid \sigma_4, (s * r)) \odot \Gamma, x : A \vdash t_2 : [\Box x/z]B\end{array}}{(\Delta \mid \sigma_3 + s * \sigma_1 \mid \sigma_4 + s * r * \sigma_1) \odot \Gamma \vdash (\mathsf{let}\,\Box x = \Box t_1\,\mathsf{in}\,t_2) = [t_1/x]t_2 : [\Box t_1/z]B}\ \textsc{Eq}_{\Box_c}$$

$$\frac{(\Delta \mid \sigma_1 \mid \sigma_2) \odot \Gamma \vdash t : \Box_s A}{(\Delta \mid \sigma_1 \mid \sigma_2) \odot \Gamma \vdash t = (\mathsf{let}\,\Box x = t\,\mathsf{in}\,\Box x) : \Box_s A}\ \textsc{Eq}_{\Box_u}$$

A subtyping relation $((\Delta \mid \sigma) \odot \Gamma \vdash A \leq B)$ subsumes equality, adding ordering of universe levels. *Type conversion* allows re-typing terms based on the judgment:

$$\frac{(\Delta \mid \sigma_1 \mid \sigma_2) \odot \Gamma \vdash t : A \quad (\Delta \mid \sigma_2) \odot \Gamma \vdash A \leq B}{(\Delta \mid \sigma_1 \mid \sigma_2) \odot \Gamma \vdash t : B}\ \textsc{Conv}$$

The full rules for equality and subtyping are in this paper's extended version [44].

## 2.4 Operational Semantics

As with other graded modal calculi (e.g., [3,10,23]), the core calculus of GRTT has a Call-by-Name small-step operational semantics with reductions $t \rightsquigarrow t'$. The rules are standard, with the addition of the $\beta$-rule for the graded modality:

$$\mathsf{let}\,\Box x = \Box t_1\,\mathsf{in}\,t_2 \rightsquigarrow [t_1/x]t_2 \qquad (\beta\Box)$$

Type preservation and normalisation are considered in Section 4.

## 2.5 Implementation and Examples

To explore our theory, we provide an implementation, **Gerty**. Section 5 describes how the declarative definition of the type theory is implemented as a bidirectional type checking algorithm. We briefly mention the syntax here for use in later examples. The following is the polymorphic identity function in **Gerty**:

```
id : (a : (.0, .2) Type 0) -> (x : (.1, .0) a) -> a
id = \a -> \x -> x
```

The syntax resembles the theory, where grading terms `.n` are syntactic sugar for a unary encoding of grades in terms of 0 and repeated addition of 1, e.g., `.2` = `(.0 + .1) + .1`. This syntax can be used for grade terms of any semiring, which can be resolved to particular built-in semirings at other points of type checking.

The following shows first projection on (non-dependent) pairs, using the graded modality (at grade 0 here) to give fine-grained usage on compound data:

```
fst : (a : (.0, .2) Type 0) (b : (.0, .1) Type 0) -> <a * [.0] b> -> a
fst = \a b p -> case p of <x, y> -> let [z] = y in x
```

The implementation adds various built-in semirings, some syntactic sugar, and extras such as: a singleton *unit* type, extensions of the theory to semirings with a pre-ordering (discussed further in Section 6), and some implicit resolution. Anywhere a grade is expected, an underscore can be supplied to indicate that **Gerty** should try to resolve the grade implicitly. Grades may also be omitted from binders (see above in `fst`), in which case they are treated as implicits. Currently, implicits are handled by generating existentially quantified grade variables, and using SMT to solve the necessary constraints (see Section 5).

So far we have considered the natural numbers semiring providing an analysis of usage. We come back to this and similar examples in Section 3. To show another kind of example, we consider a lattice semiring of privacy levels (appearing elsewhere [3,23,46]) which enforces information-flow control, akin to DCC [1]. Differently to DCC, dataflow is tracked through variable dependencies, rather than through the results of computations in the monadic style of DCC.

**Definition 1.** [Security levels] Let $\mathcal{R} = \texttt{Lo} \leq \texttt{Hi}$ be a set of labels with $0 = \texttt{Hi}$ and $1 = \texttt{Lo}$, semiring addition as the meet and multiplication as join. Here, $1 = \texttt{Lo}$ treats the base notion of dataflow as being in the low security (public) domain. Variables graded with `Hi` must then be unused, or guarded by a graded modality. This semiring is primitive in **Gerty**; we can express the following example:

```
idLo : (a : (.0, .2) Type 0) -> (x : (Lo, Hi) a) -> a
idLo = \a -> \x -> x
-- The following is rejected as ill-typed
leak : (a : (.0, .2) Type 0) -> (x : (Hi, Hi) a) -> a
leak = \a -> \x -> idLo a x
```

The first definition is well-typed, but the second yields a typing error originating from the application in its body:

```
At subject stage got the following mismatched grades:
 For 'x' expected Hi but got .1
```

where grade `1` is `Lo` here. Thus we can use this abstract label semiring as a way of restricting flow of data between regions (*cf.* region typing systems [31,55]). Note that the ordering is not leveraged here other than in the lattice operations.

## 3 Case Studies

We now demonstrate GRTT via several cases studies that focus the reasoning power of dependent types via grading. Since grading in GRTT serves to explain dataflow, we can characterise subsets of GRTT that correspond to various type theories. We demonstrate the approach with simple types, parametric polymorphism, and linearity. In each case study, we restrict GRTT to a subset by *a*

*characterisation of the grades*, rather than by, say, placing detailed syntactic restrictions or employing meta-level operations or predicates that restrict syntax (as one might do for example to map a subset of Martin-Löf type theory into the simply-typed $\lambda$-calculus by restriction to closed types, requiring deep inspection of type terms). Since this restriction is only on grades, we can harness the specific reasoning power of particular calculi from within the language itself, simply by specifications on grades. In the context of an implementation like **Gerty**, this amounts to using type signatures to restrict dataflow.

This section shows the power of tracking dataflow in types via grades, going beyond QTT [6] and GraD [13]. For 'quantitative' semirings, a 0 type-grade means that we can recover simply-typed reasoning (Section 3.3) and distinguish computational functions from type-parameter functions for parametric reasoning (Section 3.4), embedding a grade-restricted subset of GRTT into System F.

Section 5 returns to a case study that builds on the implementation.

### 3.1 Recovering Martin-Löf Type Theory

When the semiring parameterising GRTT is the singleton semiring (i.e., any semiring where $1 = 0$), we have an isomorphism $\Box_r A \cong A$, and grade annotations become redundant, as all grades are equal. All vectors and grades on binders may then be omitted, and we can write typing judgments as $\Gamma \vdash t : A$, giving rise to a standard Martin-Löf type theory as a special case of GRTT.

### 3.2 Determining Usage via Quantitative Semirings

Unlike existing systems, we can use the fine-grained grading to *guarantee* the relevance or irrelevance of assumptions in types. To do this we must consider a subset of semirings $(\mathcal{R}, *, 1, +, 0)$ called *quantitative* semirings, satisfying:

$$\begin{array}{rl} \text{(zero-unique)} & 1 \neq 0; \\ \text{(positivity)} & \forall r, s.\ r + s = 0 \implies r = 0 \wedge s = 0; \\ \text{(zero-product)} & \forall r, s.\ r * s = 0 \implies r = 0 \vee s = 0. \end{array}$$

These axioms[4] ensure that a 0-grade in a quantitative semiring represents irrelevant variable use. This notion has recently been proved for computational use by Choudhury et al. [13] via a heap-based semantics for grading (on computations) and the same result applies here. Conversely, in a quantitative semiring any grade other than 0 denotes relevance. From this, we can *directly* encode non-dependent tensors and arrows: in $(x :_0 A) \otimes B$ the grade 0 captures that $x$ cannot have any computational content in $B$, and likewise for $(x :_{(s,0)} A) \to B$ the grade 0 explains that $x$ cannot have any computational content in $B$, but may have computational use according to $s$ in the inhabiting function. Thus,

[4] Atkey requires *positivity* and *zero-product* for all semirings parameterising QTT [6] (as does Abel [2]). Atkey imposes this for admissibility of substitution. We need not place this restriction on GRTT to have substitution in general (Sec. 4.1).

the grade 0 here describes that elimination forms *cannot* ever inspect the variable during normalisation. Additionally, quantitative semirings can be used for encoding simply-typed and polymorphic reasoning.

*Example 1.* Some quantitative semirings are:

- (*Exact usage*) $(\mathbb{N}, \times, 1, +, 0)$;
- (*0-1*) The semiring over $\mathcal{R} = \{0, 1\}$ with $1 + 1 = 1$ which describes relevant *vs.* irrelevant dependencies, but no further information.
- (*None-One-Tons* [43]) The semiring on $\mathcal{R} = \{0, 1, \infty\}$ is more fine-grained than 0-1, where $\infty$ represents more than 1 usage, with $1 + 1 = \infty = 1 + \infty$.

### 3.3 Simply-typed Reasoning

As discussed in Section 1, the simply-typed $\lambda$-calculus (STLC) can be distinguished from dependently-typed calculi via the restriction of dataflow: in simple types, data can only flow at the computational level, with no dataflow within, into, or from types. We can thus view a GRTT function as simply typed when its variable is irrelevant in the type, e.g., $(x :_{(s,0)} A) \to B$ for quantitative semirings. We define a subset of GRTT restricted to simply-typed reasoning:

**Definition 2.** [Simply-typed GRTT] For a quantitative semiring, the following predicate $\textsc{Stlc}(-)$ determines a subset of simply-typed GRTT programs:

$$\textsc{Stlc}((\emptyset \mid \emptyset \mid \emptyset) \odot \emptyset \vdash t : A)$$

$$\textsc{Stlc}((\Delta \mid \sigma_1 \mid \sigma_2) \odot \Gamma \vdash t : A) \implies \textsc{Stlc}((\Delta, \mathbf{0} \mid \sigma_1, s \mid \sigma_2, 0) \odot \Gamma, x : B \vdash t : A)$$

That is, all subject-type grades are 0 (thus function types are of the form $(x :_{(s,0)} A) \to B$). A similar predicate is defined on well-formed contexts (elided), restricting context grades of well-formed contexts to only zero grading vectors.

Under the restriction of Definition 2, a subset of GRTT terms embeds into the simply-typed $\lambda$-calculus in a sound and complete way. Since STLC does not have a notion of tensor or modality, this is omitted from the encoding:

$$[\![x]\!] = x \quad [\![\lambda x.t]\!] = \lambda x.[\![t]\!] \quad [\![t_1\, t_2]\!] = [\![t_1]\!][\![t_2]\!] \quad [\![(x :_{(s,0)} A) \to B]\!]_\tau = [\![A]\!]_\tau \to [\![B]\!]_\tau$$

Variable contexts of GRTT are interpreted by point-wise applying $[\![-]\!]_\tau$ to typing assumptions. We then get the following preservation of typing into the simply-typed $\lambda$-calculus, and soundness and completeness of this encoding:

**Lemma 3 (Soundness of typing).** *Given a derivation of* $(\Delta \mid \sigma_1 \mid \sigma_2) \odot \Gamma \vdash t : A$ *such that* $\textsc{Stlc}((\Delta \mid \sigma_1 \mid \sigma_2) \odot \Gamma \vdash t : A)$ *then* $[\![\Gamma]\!]_\tau \vdash [\![t]\!] : [\![A]\!]_\tau$ *in STLC.*

**Theorem 1 (Soundness and completeness of the embedding).** *Given* $\textsc{Stlc}((\Delta \mid \sigma_1 \mid \sigma_2) \odot \Gamma \vdash t : A)$ *and* $[\![(\Delta \mid \sigma_1 \mid \sigma_2) \odot \Gamma \vdash t : A]\!]$ *then for CBN reduction* $\rightsquigarrow^{\text{STLC}}$ *in simply-typed* $\lambda$*-calculus:*

$$\begin{array}{ll} \textit{(soundness)}\ \forall t'.\ \textit{if}\ t \rightsquigarrow t' & \textit{then}\ [\![t]\!] \rightsquigarrow^{\text{STLC}} [\![t']\!] \\ \textit{(completeness)}\ \forall t_a.\ \textit{if}\ [\![t]\!] \rightsquigarrow^{\text{STLC}} t_a & \textit{then}\ \exists t'.\ t \rightsquigarrow t' \ \wedge\ [\![t']\!] \equiv_{\beta\eta} t_a \end{array}$$

Thus, we capture simply-typed reasoning just by restricting type grades to 0 for quantitative semirings. We consider quantitative semirings again for parametric reasoning, but first recall issues with parametricity and dependent types.

### 3.4 Recovering Parametricity via Grading

One powerful feature of grading in a dependent type setting is the ability to recover parametricity from dependent function types. Consider the following type of functions in System F (we borrow this example from Nuyts et al. [45]):

$$\mathsf{RI}\ A\ B\ \triangleq\ \forall\gamma.(\gamma \to A) \to (\gamma \to B)$$

Due to parametricity, we get the following notion of *representation independence* in System F: for a function $f : \mathsf{RI}\ A\ B$, some type $\gamma'$, and terms $h : \gamma' \to A$ and $c : \gamma'$, then we know that $f$ can only use $c$ by applying $h\ c$. Subsequently, $\mathsf{RI}\ A\ B \cong A \to B$ by parametricity [52], defined uniquely as:

$$\begin{array}{ll} \mathit{iso} : \mathsf{RI}\ A\ B \to (A \to B) & \quad \mathit{iso}^{-1} : (A \to B) \to \mathsf{RI}\ A\ B \\ \mathit{iso}\ f = f\ A\ (\mathit{id}\ A) & \quad \mathit{iso}^{-1}\ g = \Lambda\gamma.\ \lambda h.\ \lambda(c:\gamma).\ g(h\,c) \end{array}$$

In a dependently-typed language, one might seek to replace System F's universal quantifier with $\Pi$-types, i.e.

$$\mathsf{RI}'\ A\ B\ \triangleq\ (\gamma : \mathsf{Type}) \to (\gamma \to A) \to (\gamma \to B)$$

However, we can no longer reason parametrically about the inhabitants of such types (we cannot prove that $\mathsf{RI}'\ A\ B \cong A \to B$) as the free interaction of types and computational terms allows us to give the following non-parametric element of $\mathsf{RI}'\ A\ B$ over 'large' type instances:

$$\mathit{leak} = \lambda\gamma.\ \lambda h.\ \lambda c.\ \gamma : \mathsf{RI}'\ A\ \mathsf{Type}$$

Instead of applying $h\ c$, the above "leaks" the type parameter $\gamma$. Grtt can recover universal quantification, and hence parametric reasoning, by using grading to restrict the data-flow capabilities of a $\Pi$-type. We can refine representation independence to the following:

$$\mathsf{RI}''\ A\ B\ \triangleq\ (\gamma :_{(0,2)} \mathsf{Type}) \to (h :_{(s_1,0)} (x :_{(s_2,0)} \gamma) \to A) \to (c :_{(s_3,0)} \gamma) \to B$$

for some grades $s_1$, $s_2$, and $s_3$, and with shorthand $2 = 1 + 1$.

If we look at the definition of *leak* above, we see that $\gamma$ is used in the body of the function and thus requires usage 1, so *leak* cannot inhabit $\mathsf{RI}''\ A\ \mathsf{Type}$. Instead, *leak* would be typed differently as:

$$\mathit{leak} : (\gamma :_{(1,2)} \mathsf{Type}) \to (h :_{(0,0)} (x :_{(s,0)} \gamma) \to A) \to (c :_{(0,0)} \gamma) \to \mathsf{Type}$$

The problematic behaviour (that the type parameter $\gamma$ is returned by the inner function) is exposed by the subject grade 1 on the binder of $\gamma$. We can thus define a graded universal quantification from a graded $\Pi$-typed:

$$\forall_r(\gamma : A).B \triangleq (\gamma :_{(0,r)} A) \to B \tag{1}$$

This denotes that the type parameter $\gamma$ can appear freely in $B$ described by grade $r$, but is irrelevant in the body of any corresponding $\lambda$-abstraction. This is akin to the work of Nuyts et al. who develop a system with several modalities for regaining parametricity within a dependent type theory [45]. Note however that parametricity is recovered for us here as one of many possible options coming from systematically specialising the grading.

*Capturing Existential Types* With the ability to capture universal quantifier, we can similarly define existentials (allowing, e.g., abstraction [11]). We define the existential type via a Church-encoding as follows:

$$\exists_{r}(x : A).B \triangleq \forall_{2}(C : \mathsf{Type}_l).(f :_{(1,0)} \forall_{r}(x : A).(b :_{(s,0)} B) \to C) \to C$$

*Embedding into Stratified System F* We show that parametricity is regained here (and thus eqn. (1) really behaves as a universal quantifier and not a general $\Pi$-type) by showing that we can embed a subset of GRTT into System F, based solely on a classification of the grades. We follow a similar approach to Section 3.3 for simply-typed reasoning but rather than defining a purely syntactic encoding (and then proving it type sound) our encoding is type directed since we embed GRTT functions of type $(x :_{(0,r)} \mathsf{Type}_l) \to B$ as universal types in System F with corresponding type abstractions ($\Lambda$) as their inhabitants. Since GRTT employs a predicative hierarchy of universes, we target Stratified System F (hereafter SSF) since it includes the analogous inductive hierarchy of kinds [38]. We use the formulation of Eades and Stump [21] with terms $t_s$ and types $T$:

$$t_s ::= x \mid \lambda(x : T).t_s \mid t_s\, t'_s \mid \Lambda(X : K).t_s \mid t_s\,[T] \qquad T ::= X \mid T \to T' \mid \forall(X : K).T$$

with kinds $K ::= \star_l$ where $l \in \mathbb{N}$ providing the stratified kind hierarchy. Capitalised variables $X$ are System F type variables and $t_s\,[T]$ is type application. Contexts may contain both type and computational variables, and so free-variable type assumptions may have dependencies, akin to dependent type systems. Kinding is via judgments $\Gamma \vdash T : \star_l$ and typing via $\Gamma \vdash t : T$.

We define a type directed encoding on a subset of GRTT typing derivations characterised by the following predicate:

$$\begin{aligned}
&\textsc{Ssf}((\emptyset \mid \emptyset \mid \emptyset) \odot \emptyset \vdash t : A)\\
&\textsc{Ssf}((\Delta \mid \sigma_1 \mid \sigma_2) \odot \Gamma \vdash t : A) \implies \textsc{Ssf}((\Delta, \mathbf{0} \mid \sigma_1, 0 \mid \sigma_2, r) \odot \Gamma, x : \mathsf{Type}_l \vdash t : A)\\
&\textsc{Ssf}((\Delta \mid \sigma_1 \mid \sigma_2) \odot \Gamma \vdash t : A) \wedge \mathsf{Type}_l \not\in^{+ve} B\\
&\qquad\qquad \implies \textsc{Ssf}((\Delta, \sigma_3 \mid \sigma_1, s \mid \sigma_2, 0) \odot \Gamma, x : B \vdash t : A)
\end{aligned}$$

By $\mathsf{Type}_l \not\in^{+ve} B$ we mean $\mathsf{Type}_l$ is not a positive subterm of $B$, avoiding higher-order typing terms (e.g., type constructors) which do not exist in SSF.

Under this restriction, we give a type-directed encoding mapping derivations of GRTT to SSF: given a GRTT derivation of judgment $(\Delta \mid \sigma_1 \mid \sigma_2) \odot \Gamma \vdash t : A$ we have that $\exists t_s$ (an SSF term) such that there is a derivation of judgment $[\![\Gamma]\!] \vdash t_s : [\![A]\!]_\tau$ in SSF where we interpret a subset of GRTT terms $A$ as types:

$$\begin{aligned}
[\![x]\!]_\tau &= x\\
[\![\mathsf{Type}_l]\!]_\tau &= \star_l\\
[\![(x :_{(0,r)} \mathsf{Type}_l) \to B]\!]_\tau &= \forall x : \star_l.[\![B]\!]_\tau && \textit{where } \mathsf{Type}_l \not\in^{+ve} B\\
[\![(x :_{(s,0)} A) \to B]\!]_\tau &= [\![A]\!]_\tau \to [\![B]\!]_\tau && \textit{where } \mathsf{Type}_l \not\in^{+ve} A, B
\end{aligned}$$

Thus, dependent functions with $\mathsf{Type}$ parameters that are computationally irrelevant (subject grade 0) map to $\forall$ types, and dependent functions with parameters irrelevant in types (subject-type grade 0) map to regular function types.

We elide the full details but sketch key parts where functions and applications are translated inductively (where $\mathsf{Ty}_l$ is shorthand for $\mathsf{Type}_l$):

$$[\![\frac{(\Delta, \sigma_1 \mid \sigma_2, 0 \mid \sigma_3, r) \odot \Gamma, x : \mathsf{Ty}_l \vdash t : B}{(\Delta \mid \sigma_2 \mid \sigma_1 + \sigma_3) \odot \Gamma \vdash \lambda x.t : (x :_{(0,r)} \mathsf{Ty}_l) \to B}]\!] = \frac{[\![\Gamma]\!], x : \star_l \vdash t_s : [\![B]\!]_\tau}{[\![\Gamma]\!] \vdash \Lambda(x : \star_l).t_s : \forall x : \star_l.[\![B]\!]_\tau}$$

$$[\![\frac{(\Delta, \sigma_1 \mid \sigma_2, s \mid \sigma_3, 0) \odot \Gamma, x : A \vdash t : B}{(\Delta \mid \sigma_2 \mid \sigma_1 + \sigma_3) \odot \Gamma \vdash \lambda x.t : (x :_{(s,0)} A) \to B}]\!] = \frac{[\![\Gamma]\!], x : [\![A]\!]_\tau \vdash t_s : [\![B]\!]_\tau}{[\![\Gamma]\!] \vdash \lambda(x : [\![A]\!]_\tau).t_s : [\![A]\!]_\tau \to [\![B]\!]_\tau}$$

$$[\![\frac{\begin{array}{l}(\Delta \mid \sigma_2 \mid \sigma_1 + \sigma_3) \odot \Gamma \vdash t_1 : (x :_{(0,r)} \mathsf{Ty}_l) \to B \\ (\Delta \mid \sigma_4 \mid \sigma_1) \odot \Gamma \vdash t_2 : \mathsf{Ty}_l\end{array}}{(\Delta \mid \sigma_2 \mid \sigma_3 + r * \sigma_4) \odot \Gamma \vdash t_1\, t_2 : [t_2/x]B}]\!] = \frac{\begin{array}{l}[\![\Gamma]\!] \vdash t_s : \forall(x : \star_l).[\![B]\!]_\tau \\ [\![\Gamma]\!] \vdash T : \star_l\end{array}}{[\![\Gamma]\!] \vdash t_s[T] : [T/x][\![B]\!]_\tau}$$

$$[\![\frac{\begin{array}{l}(\Delta \mid \sigma_2 \mid \sigma_1 + \sigma_3) \odot \Gamma \vdash t_1 : (x :_{(s,0)} A) \to B \\ (\Delta \mid \sigma_4 \mid \sigma_1) \odot \Gamma \vdash t_2 : A\end{array}}{(\Delta \mid \sigma_2 + s * \sigma_4 \mid \sigma_3) \odot \Gamma \vdash t_1\, t_2 : [t_2/x]B}]\!] = \frac{\begin{array}{l}[\![\Gamma]\!] \vdash t_s : [\![A]\!]_\tau \to [\![B]\!]_\tau \\ [\![\Gamma]\!] \vdash t'_s : [\![A]\!]_\tau\end{array}}{[\![\Gamma]\!] \vdash t_s\; t'_s : [t'_s/x][\![B]\!]_\tau}$$

In the last case, note the presence of $[t'_s/x][\![B]\!]_\tau$. Reasoning under the context of the encoding, this is proven equivalent to $[\![B]\!]_\tau$ since the subject type grade is 0 and therefore use of $x$ in $B$ is irrelevant.

**Theorem 2 (Soundness and completeness of SSF embedding).** *Given* $\textsc{Ssf}((\Delta \mid \sigma_1 \mid \sigma_2) \odot \Gamma \vdash t : A)$ *and* $t_a$ *in SSF where* $[\![(\Delta \mid \sigma_1 \mid \sigma_2) \odot \Gamma \vdash t : A]\!] = [\![\Gamma]\!] \vdash t_s : [\![A]\!]_\tau$ *then for CBN reduction* $\leadsto^{\textsc{Ssf}}$ *in Stratified System F:*

$$\begin{array}{rl}\textit{(soundness)}\ \forall t'.\ t \leadsto t' & \implies \exists t'_s.t_s \leadsto^{\text{SSF}} t'_s \\ & \quad \wedge\ [\![(\Delta \mid \sigma_1 \mid \sigma_2) \odot \Gamma \vdash t' : A]\!] = [\![\Gamma]\!] \vdash t'_s : [\![A]\!]_\tau \\ \textit{(completeness)}\ \forall t'_s.\ t_s \leadsto^{\text{SSF}} t'_s & \implies \exists t'.t \leadsto t' \\ & \quad \wedge\ [\![(\Delta \mid \sigma_1 \mid \sigma_2) \odot \Gamma \vdash t' : A]\!] = [\![\Gamma]\!] \vdash t'_s : [\![A]\!]_\tau\end{array}$$

Thus, we can capture parametricity in GRTT via the judicious use of 0 grading (at either the type or computational level) for quantitative semirings. This embedding is not possible from QTT since QTT variables graded with 0 may be used arbitrarily in the types; the embedding here relies on GRTT's 0 type-grade capturing abscence in types for quantitative semirings.

### 3.5 Graded Modal Types and Non-dependent Linear Types

GRTT can embed the reasoning present in other graded modal type theories (which often have a linear base), for example the explicit semiring-graded necessity modality found in coeffect calculi [10,23] and Granule [46]. We can recover the axioms of a graded necessity modality (usually modelled by an exponential graded comonad [23]). For example, in **Gerty** the following are well typed:

```
counit : (a : (.0, .2) Type) -> (z : (.1 , .0) [.1] a) -> a
counit = \a z -> case z of [y] -> y
comult : (a : (.0, .2) Type) -> (z : (.1 , .0) [.6] a) -> [.2] ([.3] a)
comult = \a z -> case z of [y] -> [[y]]
```

corresponding to $\varepsilon : \Box_1 A \to A$ and $\delta_{r,s} : \Box_{r*s} A \to \Box_r(\Box_s A)$: operations of graded necessity / graded comonads. Since we cannot use arbitrary terms for grades in the implementation, we have picked some particular grades here for `comult`. First-class grading is future work, discussed in Section 6.

Linear functions can be captured as $A \multimap B \triangleq (x :_{(1,r)} A) \to B$ for an exact usage semiring. It is straightforward to characterise a subset of GRTT programs that maps to the linear $\lambda$-calculus akin to the encodings above. Thus, GRTT provides a suitable basis for studying both linear and non-linear theories alike.

## 4 Metatheory

We now study GRTT's metatheory. We first explain how substitution presents itself in the theory, and how type preservation follows from a relationship between equality and reduction. We then show admissibility of graded structural rules for contraction, exchange, and weakening, and strong normalization.

### 4.1 Substitution

We introducing substitution for well-formed contexts and then typing.

**Lemma 4 (Substitution for well-formed contexts).** *If the following hold:*

*1.* $(\Delta \mid \sigma_2 \mid \sigma_1) \odot \Gamma_1 \vdash t : A$ *and* *2.* $(\Delta, \sigma_1, \Delta') \odot \Gamma_1, x : A, \Gamma_2 \vdash$

*Then:* $\Delta, (\Delta' \backslash |\Delta| + (\Delta' / |\Delta|) * \sigma_2) \odot \Gamma_1, [t/x]\Gamma_2 \vdash$

That is, given $\Gamma_1, x : A, \Gamma_2$ is well-formed, we can cut out $x$ by substituting $t$ for $x$ in $\Gamma_2$, accounting for the new usage in the context grade vectors. The usage of $\Gamma_1$ in $t$ is given by $\sigma_2$, and the usage in $A$ by $\sigma_1$. When substituting, $\Delta$ remains the same, as $\Gamma_1$ is unchanged. However, to account for the usage in $[t/x]\Gamma_2$, we have to form a new context grade vector $\Delta' \backslash |\Delta| + (\Delta' / |\Delta|) * \sigma_2$.

The operation $\Delta' \backslash |\Delta|$ (pronounced 'discard') removes grades corresponding to $x$, by removing the grade at index $|\Delta|$ from each grade vector in $\Delta'$. Everything previously used in the typing of $x$ in the context must now be distributed across $[t/x]\Gamma_2$, which is done by adding on $(\Delta' / |\Delta|) * \sigma_2$, which uses $\Delta' / |\Delta|$ (pronounced 'choose') to produce a vector of grades, which correspond to the grades cut out in $\Delta' \backslash |\Delta|$. The multiplication of $(\Delta' / |\Delta|) * \sigma_2$ produces a context grade vector by scaling $\sigma_2$ by each element of $(\Delta' / |\Delta|)$. When adding vectors, if the sizes of the vectors are different, then the shorter vector is right-padded with zeroes. Thus $\Delta' \backslash |\Delta| + (\Delta' / |\Delta|) * \sigma_2$ can be read as '*$\Delta'$ without the grades corresponding to $x$, plus the usage of $t$ scaled by the prior usage of $x$*'.

For example, given typing $((), (1) \mid 0, 1 \mid 1, 0) \odot a : \mathsf{Type}, y : a \vdash y : a$ and well-formed context $((), (1), (1, 0), (0, 0, 2)) \odot a : \mathsf{Type}, y : a, x : a, z : t' \vdash$, where $t'$ uses $x$ twice, we can substitute $y$ for $x$. Therefore, let $\Gamma_1 = a : \mathsf{Type}, y : a$ thus $|\Gamma_1| = 2$ and $\Gamma_2 = z : x$ and $\Delta' = ((0, 0, 2))$ and $\sigma_1 = 1, 0$ and $\sigma_2 = 0, 1$. Then the context grade of the substitution $[y/x]\Gamma_2$ is calculated as:

$$((0, 0, 2)) \backslash |\Gamma_1| = ((0, 0)) \qquad (((0, 1, 2)) / |\Gamma_1|) * \sigma_2 = (2) * (0, 1) = ((0, 2))$$

Thus the resulting judgment is $((), (1), (0, 2)) \odot a : \mathsf{Type}, y : a, z : [y/x]t' \vdash$.

**Lemma 5 (Substitution for typing).** *If the following premises hold:*

1. $(\Delta \mid \sigma_2 \mid \sigma_1) \odot \Gamma_1 \vdash t : A$
2. $(\Delta, \sigma_1, \Delta' \mid \sigma_3, s, \sigma_4 \mid \sigma_5, r, \sigma_6) \odot \Gamma_1, x : A, \Gamma_2 \vdash t' : B$
3. $|\sigma_3| = |\sigma_5| = |\Gamma_1|$

*Then* $\begin{pmatrix} \Delta,(\Delta' \backslash |\Delta| + (\Delta'/|\Delta|) * \sigma_2) \\ (\sigma_3 + s * \sigma_2), \sigma_4 \\ (\sigma_5 + r * \sigma_2), \sigma_6 \end{pmatrix} \odot \Gamma_1, [t/x]\Gamma_2 \vdash [t/x]t' : [t/x]B$.

As with substitution for well-formed contexts, we account for the replacement of $x$ with $t$ in $\Gamma_2$ by 'cutting out' $x$ from the context grade vectors, and adding on the grades required to form $t$, scaled by the grades that described $x$'s usage. We additionally must account for the altered subject and subject-type usage. We do this in a similar manner, by taking, for example, the usage of $\Gamma_1$ in the subject ($\sigma_3$), and adding on the grades required to form $t$, scaled by the grade with which $x$ was previously used ($s$). Subject-type grades are calculated similarly.

## 4.2 Type Preservation

**Lemma 6.** *Reduction implies equality If* $(\Delta \mid \sigma_1 \mid \sigma_2) \odot \Gamma \vdash t_1 : A$ *and* $t_1 \rightsquigarrow t_2$*, then* $(\Delta \mid \sigma_1 \mid \sigma_2) \odot \Gamma \vdash t_1 = t_2 : A$.

**Lemma 7.** *Equality inversion If* $(\Delta \mid \sigma_1 \mid \sigma_2) \odot \Gamma \vdash t_1 = t_2 : A$*, then* $(\Delta \mid \sigma_1 \mid \sigma_2) \odot \Gamma \vdash t_1 : A$ *and* $(\Delta \mid \sigma_1 \mid \sigma_2) \odot \Gamma \vdash t_2 : A$.

**Lemma 8.** *Type preservation If* $(\Delta \mid \sigma_1 \mid \sigma_2) \odot \Gamma \vdash t : A$ *and* $t \rightsquigarrow t'$*, then* $(\Delta \mid \sigma_1 \mid \sigma_2) \odot \Gamma \vdash t' : A$.

*Proof.* By Lemma 6 we have $(\Delta \mid \sigma_1 \mid \sigma_2) \odot \Gamma \vdash t = t' : A$, and therefore by Lemma 7 we have $(\Delta \mid \sigma_1 \mid \sigma_2) \odot \Gamma \vdash t' : A$, as required.

## 4.3 Structural Rules

We now consider the structural rules of *contraction*, *exchange*, and *weakening*.

**Lemma 9 (Contraction).** *The following rule is admissible:*

$$\frac{\begin{pmatrix} \Delta_1, \sigma_1, (\sigma_1, 0), \Delta_2 \\ \sigma_2, s_1, s_2, \sigma_3 \\ \sigma_4, r_1, r_2, \sigma_5 \end{pmatrix} \odot \Gamma_1, x : A, y : A, \Gamma_2 \vdash t : B \quad |\Delta_1| = |\sigma_2| = |\sigma_4| = |\Gamma_1|}{\begin{pmatrix} \Delta_1, \sigma_1, \mathsf{contr}(|\Delta_1|; \Delta_2) \\ \sigma_2, (s_1 + s_2), \sigma_3 \\ \sigma_4, (r_1 + r_2), \sigma_5 \end{pmatrix} \odot \Gamma_1, z : A, [z, z/x, y]\Gamma_2 \vdash [z, z/x, y]t : [z, z/x, y]B} \textsc{Contr}$$

The operation $\mathsf{contr}(\pi; \Delta)$ contracts the elements at index $\pi$ and $\pi + 1$ for each vector in $\Delta$ by combining them with the semiring addition, defined $\mathsf{contr}(\pi; \Delta) = \Delta \backslash (\pi + 1) + \Delta/(\pi + 1) * (\mathbf{0}^{\pi}, 1)$. Admissibility follows from the semiring addition, which serves to contract dependencies, being threaded throughout the rules.

**Lemma 10 (Exchange).** *The following rule is admissible:*

$$\frac{x \notin \mathsf{FV}(B) \qquad |\Delta_1| = |\sigma_3| = |\sigma_5| = |\Gamma_1| \qquad \left(\begin{smallmatrix}\Delta_1,\sigma_1,(\sigma_2,0),\Delta_2\\ \sigma_3,s_1,s_2,\sigma_4\\ \sigma_5,r_1,r_2,\sigma_6\end{smallmatrix}\right) \odot \Gamma_1, x : A, y : B, \Gamma_2 \vdash t : C}{\left(\begin{smallmatrix}\Delta_1,\sigma_2,(\sigma_1,0),\mathsf{exch}(|\Delta_1|;\Delta_2)\\ \sigma_3,s_2,s_1,\sigma_4\\ \sigma_5,r_2,r_1,\sigma_6\end{smallmatrix}\right) \odot \Gamma_1, y : B, x : A, \Gamma_2 \vdash t : C}\ \textsc{Exc}$$

Notice that if you strip away the vector fragment and sizing premise, this is exactly the form of exchange we would expect in a dependent type theory: if $x$ and $y$ are assumptions in a context typing $t : C$, and the type of $y$ does not depend upon $x$, then we can type $t : C$ when we swap the order of $x$ and $y$.

The action on grade vectors is simple: we swap the grades associated with each of the variables. For the context grade vector however, we must do two things: first, we capture the formation of $A$ with $\sigma_1$, and the formation of $B$ with $\sigma_1, 0$ (indicating $x$ being used with grade 0 in $B$), then swap these around, cutting the final grade from $\sigma_2, 0$, and adding 0 to the end of $\sigma_1$ to ensure correct sizing. Next, the operation $\mathsf{exch}(|\Delta_1|\,; \Delta_2)$ swaps the element at index $|\Delta_1|$ (i.e., that corresponding to usage of $x$) with the element at index $|\Delta_1| + 1$ (corresponding to $y$) for every vector in $\Delta_2$; this exchange operation ensures that usage in the trailing context is reordered appropriately.

**Lemma 11 (Weakening).** *The following rule is admissible:*

$$\frac{(\Delta_1, \Delta_2 \mid \sigma_1, \sigma_1' \mid \sigma_2, \sigma_2') \odot \Gamma_1, \Gamma_2 \vdash t : B \qquad (\Delta_1 \mid \sigma_3 \mid \mathit{0}) \odot \Gamma_1 \vdash A : \mathsf{Type}_l \qquad |\sigma_1| = |\sigma_2| = |\Gamma_1|}{(\Delta_1, \sigma_3, \mathsf{ins}(|\Delta_1|\,; 0; \Delta_2) \mid \sigma_1, 0, \sigma_1' \mid \sigma_2, 0, \sigma_2') \odot \Gamma_1, x : A, \Gamma_2 \vdash t : B}\ \textsc{Weak}$$

Weakening introduces irrelevant assumptions to a context. We do this by capturing the usage in the formation of the assumption's type with $\sigma_3$ to preserve the well-formedness of the context. We then indicate irrelevance of the assumption by grading with 0 in appropriate places. The operation $\mathsf{ins}(\pi; s; \Delta)$ inserts the element $s$ at index $\pi$ for each $\sigma$ in $\Delta$, such that all elements preceding index $\pi$ (in $\sigma$) keep their positions, and every element at index $\pi$ or greater (in $\sigma$) will be shifted one index later in the new vector. The 0 grades in the subject and subject-type grade vector positions correspond to the absence of the irrelevant assumption from the subject and subject's type.

### 4.4 Strong Normalization

We adapt Geuvers' strong normalization proof for the Calculus of Constructions (CC) [24] to a fragment of GRTT (called GRTT$^{\{0,1\}}$) restricted to two universe levels and without variables of type $\mathsf{Type}_1$. This results in a less expressive system than full GRTT when it comes to higher kinds, but this is orthogonal to the main idea here of grading. We briefly overview the strong normalization proof; details can be found in the extended version [44]. Note this strong normalization result is with respect to $\beta$-reduction only (our semantics does not include $\eta$-reduction).

We use the proof technique of saturated sets, based on the reducibility candidates of Girard [29]. While GRTT$^{\{0,1\}}$ has a collapsed syntax we use judgments to break typing up into stages. We use these sets to match on whether a term is a kind, type, constructor, or a function (we will refer to these as terms).

**Definition 3.** Typing can be broken up into the following stages:

$$\begin{array}{lcl}
\mathsf{Kind} & := & \{A \mid \exists \Delta, \sigma_1, \Gamma.(\Delta \mid \sigma_1 \mid \mathbf{0}) \odot \Gamma \vdash A : \mathsf{Type}_1\} \\
\mathsf{Type} & := & \{A \mid \exists \Delta, \sigma_1, \Gamma.(\Delta \mid \sigma_1 \mid \mathbf{0}) \odot \Gamma \vdash A : \mathsf{Type}_0\} \\
\mathsf{Con} & := & \{t \mid \exists \Delta, \sigma_1, \sigma_2, \Gamma, A.(\Delta \mid \sigma_1 \mid \sigma_2) \odot \Gamma \vdash t : A \wedge (\Delta \mid \sigma_2 \mid \mathbf{0}) \odot \Gamma \vdash A : \mathsf{Type}_1\} \\
\mathsf{Term} & := & \{t \mid \exists \Delta, \sigma_1, \sigma_2, \Gamma, A.(\Delta \mid \sigma_1 \mid \sigma_2) \odot \Gamma \vdash t : A \wedge (\Delta \mid \sigma_2 \mid \mathbf{0}) \odot \Gamma \vdash A : \mathsf{Type}_0\}
\end{array}$$

**Lemma 12 (Classification).** *We have* $\mathsf{Kind} \cap \mathsf{Type} = \emptyset$ *and* $\mathsf{Con} \cap \mathsf{Term} = \emptyset$.

The classification lemma states that we can safely case split over kinds and types, or constructors and terms without fear of an overlap occurring.

Saturated sets are essentially collections of strongly normalizing terms that are closed under $\beta$-reduction. The intuition behind this proof is that every typable program ends up in some saturated set, and hence, is strongly normalizing.

**Definition 4.** [Base terms and saturated terms] Informally, the set of base terms $\mathcal{B}$ is inductively defined from variables and $\mathsf{Type}_0$ and $\mathsf{Type}_1$, and compound terms over base $\mathcal{B}$ and strongly normalising terms $\mathsf{SN}$.

A set of terms $X$ is *saturated* if $X \subset \mathsf{SN}$, $\mathcal{B} \subset X$, and if $\mathsf{red}_{\mathsf{k}}\, t \in X$ and $t \in \mathsf{SN}$, then $t \in X$. Thus saturated sets are closed under strongly normalizing terms with a *key redex*, denoted $\mathsf{red}_{\mathsf{k}}\, t$, which are redexes or a redex at the head of an elimination form. $\mathsf{SAT}$ denotes the collection of saturated sets.

**Lemma 13 ($\mathsf{SN}$ saturated).** *All saturated sets are non-empty;* $\mathsf{SN}$ *is saturated.*

Since GRTT$^{\{0,1\}}$ allows computation in types as well as in types, we separate the interpretations for kinds and types, where the former is a set of the latter.

**Definition 5.** For $A \in \mathsf{Kind}$, the kind interpretation, $\mathcal{K}[\![A]\!]$, is defined:

$$\begin{array}{ll}
\mathcal{K}[\![\mathsf{Type}_0]\!] = \mathsf{SAT} & \mathcal{K}[\![(x :_{(s,r)} A) \to B]\!] = \{f \mid f : \mathcal{K}[\![A]\!] \to \mathcal{K}[\![B]\!]\}, \text{ if } A, B \in \mathsf{Kind} \\
\mathcal{K}[\![\Box_s A]\!] \;\; = \mathcal{K}[\![A]\!] & \mathcal{K}[\![(x :_{(s,r)} A) \to B]\!] = \mathcal{K}[\![A]\!], \text{ if } A \in \mathsf{Kind}, B \in \mathsf{Type} \\
& \mathcal{K}[\![(x :_{(s,r)} A) \to B]\!] = \mathcal{K}[\![B]\!], \text{ if } A \in \mathsf{Type}, B \in \mathsf{Kind} \\
& \mathcal{K}[\![(x :_r A) \otimes B]\!] \quad = \mathcal{K}[\![A]\!] \times \mathcal{K}[\![B]\!], \text{ if } A, B \in \mathsf{Kind} \\
& \mathcal{K}[\![(x :_r A) \otimes B]\!] \quad = \mathcal{K}[\![A]\!], \text{ if } A \in \mathsf{Kind}, B \in \mathsf{Type} \\
& \mathcal{K}[\![(x :_r A) \otimes B]\!] \quad = \mathcal{K}[\![B]\!], \text{ if } A \in \mathsf{Type}, B \in \mathsf{Kind}
\end{array}$$

Next we define the interpretation of types, which requires the interpretation to be parametric on an interpretation of type variables called a type evaluation. This is necessary to make the interpretation well-founded (first realized by Girard [29]).

**Definition 6.** *Type valuations*, $\Delta \odot \Gamma \models \varepsilon$, are defined as follows:

$$\frac{}{\emptyset \odot \emptyset \models \emptyset}\,\textsc{E} \qquad \frac{X \in \mathcal{K}[\![A]\!] \qquad \Delta \odot \Gamma \models \varepsilon \qquad (\Delta \mid \sigma \mid \mathbf{0}) \odot \Gamma \vdash A : \mathsf{Type}_1}{(\Delta, \sigma) \odot (\Gamma, x : A) \models \varepsilon[x \mapsto X]}\,\textsc{Ty} \qquad \frac{\Delta \odot \Gamma \models \varepsilon \qquad (\Delta \mid \sigma \mid \mathbf{0}) \odot \Gamma \vdash A : \mathsf{Type}_0}{(\Delta, \sigma) \odot (\Gamma, x : A) \models \varepsilon}\,\textsc{Tm}$$

Type valuations ignore term variables (rule TM), in fact, the interpretations of both types and kinds ignores them because we are defining sets of terms over types, and thus terms in types do not contribute to the definition of these sets. However as these interpretations define sets of open terms we must carry a graded context around where necessary. Thus, type valuations are with respect to a well-formed graded context $\Delta \odot \Gamma$. We now outline the type interpretation.

**Definition 7.** For type valuation $\Delta \odot \Gamma \models \varepsilon$ and a type $A \in (\mathsf{Kind} \cup \mathsf{Type} \cup \mathsf{Con})$ with $A$ typable in $\Delta \odot \Gamma$, the interpretation of types $[\![A]\!]_\varepsilon$ is defined inductively. For brevity, we list just a few illustrative cases, including modalities and some function cases; the complete definition is given in the extended version [44].

$$
\begin{aligned}
[\![\mathsf{Type}_1]\!]_\varepsilon &= \mathsf{SN} \\
[\![\mathsf{Type}_0]\!]_\varepsilon &= \lambda X \in \mathsf{SAT}.\mathsf{SN} \\
[\![x]\!]_\varepsilon &= \varepsilon\, x && \text{if } x \in \mathsf{Con} \\
[\![\Box_s A]\!]_\varepsilon &= [\![A]\!]_\varepsilon \\
[\![\lambda x : A.B]\!]_\varepsilon &= \lambda X \in \mathcal{K}[\![A]\!].[\![B]\!]_{\varepsilon[x \mapsto X]} && \text{if } A \in \mathsf{Kind}, B \in \mathsf{Con} \\
[\![A\, B]\!]_\varepsilon &= [\![A]\!]_\varepsilon([\![B]\!]_\varepsilon) && \text{if } B \in \mathsf{Con} \\
[\![(x :_{(s,r)} A) \to B]\!]_\varepsilon &= \lambda X \in \mathcal{K}[\![A]\!] \to \mathcal{K}[\![B]\!]. \textstyle\bigcap_{Y \in \mathcal{K}[\![A]\!]} ([\![A]\!]_\varepsilon\, Y \to [\![B]\!]_{\varepsilon[x \mapsto Y]}\, (X\,(Y))) \\
& && \text{if } A, B \in \mathsf{Kind}
\end{aligned}
$$

Grades play no role in the reduction relation for GRTT, and hence, our interpretation erases graded modalities and their introductory and elimination forms (translated into substitutions). In fact, the above interpretation can be seen as a translation of $\text{GRTT}^{\{0,1\}}$ into non-substructural set theory; there is no data-usage tracking in the image of the interpretation. Tensors are translated into Cartesian products whose eliminators are translated into substitutions similarly to graded modalities. All terms however remain well-typed through the interpretation.

The interpretation of terms corresponds to term valuations that are used to close the term before interpreting it into the interpretation of its type.

**Definition 8.** *Valid term valuations*, $\Delta \odot \Gamma \models_\varepsilon \rho$, are defined as follows:

$$
\frac{}{\emptyset \odot \emptyset \models_\emptyset \emptyset}\,\textsc{E}
\qquad
\frac{\begin{array}{l} t \in ([\![A]\!]_\varepsilon)\,(\varepsilon\, x) \\ \Delta \odot \Gamma \models_\varepsilon \rho \\ (\Delta \mid \sigma \mid \mathbf{0}) \odot \Gamma \vdash A : \mathsf{Type}_1 \end{array}}{(\Delta, \sigma) \odot \Gamma, x : A \models_\varepsilon \rho[x \mapsto t]}\,\textsc{Ty}
\qquad
\frac{\begin{array}{l} t \in [\![A]\!]_\varepsilon \\ \Delta \odot \Gamma \models_\varepsilon \rho \\ (\Delta \mid \sigma \mid \mathbf{0}) \odot \Gamma \vdash A : \mathsf{Type}_0 \end{array}}{(\Delta, \sigma) \odot \Gamma, x : A \models_\varepsilon \rho[x \mapsto t]}\,\textsc{Tm}
$$

We interpret terms as substitutions, but graded modalities must be erased and their elimination forms converted into substitutions (and similarly for the eliminator for tensor products).

**Definition 9.** Suppose $\Delta \odot \Gamma \models_\varepsilon \rho$. Then the *interpretation of a term* $t$ typable in $\Delta \odot \Gamma$ is $(\!|t|\!)_\rho = \rho\, t$, but where all let-expressions are translated into substitutions, and all graded modalities are erased.

Finally, we prove our main result using semantic typing which will imply strong normalization. Suppose $(\Delta \mid \sigma_1 \mid \sigma_2) \odot \Gamma \vdash t : A$, then:

**Definition 10.** *Semantic typing*, $(\Delta \mid \sigma_1 \mid \sigma_2) \odot \Gamma \models t : A$, is defined as follows:

1. If $(\Delta \mid \sigma \mid \mathbf{0}) \odot \Gamma \vdash A : \mathsf{Type}_1$, then for every $\Delta \odot \Gamma \models_\varepsilon \rho$, $(\!|t|\!)_\rho \in [\![A]\!]_\varepsilon\,([\![t]\!]_\varepsilon)$.
2. If $(\Delta \mid \sigma \mid \mathbf{0}) \odot \Gamma \vdash A : \mathsf{Type}_0$, then for every $\Delta \odot \Gamma \models_\varepsilon \rho$, $(\!|t|\!)_\rho \in [\![A]\!]_\varepsilon$.

**Theorem 3 (Soundness for Semantic Typing).** $(\Delta \mid \sigma_1 \mid \sigma_2) \odot \Gamma \models t : A$.

**Corollary 1 (Strong Normalization).** *We have* $t \in \mathsf{SN}$.

## 5 Implementation

Our implementation **Gerty** is based on a bidirectionalised version of the typing rules here, somewhat following traditional schemes of bidirectional typing [19,20] but with grading (similar to Granule [46] but adapted considerably for the dependent setting). We briefly outline the implementation scheme and highlight a few key points, rules, and examples. We use this implementation to explore further applications of GRTT, namely optimising type checking algorithms.

Bidirectional typing splits declarative typing rules into *check* and *infer* modes. Furthermore, bidirectional GRTT rules split the grading context (left of $\odot$) into *input* and *output* contexts where $(\Delta \mid \sigma_1 \mid \sigma_2) \odot \Gamma \vdash t : A$ is implemented via:

$$(\mathit{check})\ \ \Delta; \Gamma \vdash t \Leftarrow A; \sigma_1; \sigma_2 \quad \mathit{or} \quad (\mathit{infer})\ \ \Delta; \Gamma \vdash t \Rightarrow A; \sigma_1; \sigma_2$$

where $\Leftarrow$ rules *check* that $t$ has type $A$ and $\Rightarrow$ rules *infer* (calculate) that $t$ has type $A$. In both judgments, the context grading $\Delta$ and context $\Gamma$ left of $\vdash$ are inputs whereas the grade vectors $\sigma_1$ and $\sigma_2$ to the right of $A$ are outputs. This input-output context approach resembles that employed in linear type checking [5,32,62]. Rather than following a "left over" scheme as in these works (where the output context explains what resources are left), the output grades here explain what has been used according to the analysis of grading ('adding up' rather than 'taking away').

For example, the following is the *infer* rule for function elimination:

$$\frac{\begin{array}{l}\Delta; \Gamma \vdash t_1 \Rightarrow (x :_{(s,r)} A) \to B; \sigma_2; \sigma_{13} \\ \Delta; \Gamma \vdash t_2 \Leftarrow A; \sigma_4; \sigma_1 \\ \Delta, \sigma_1; \Gamma, x : A \vdash B \Rightarrow \mathsf{Type}_l; \sigma_3, r; \mathbf{0} \qquad \sigma_{13} = \sigma_1 + \sigma_3\end{array}}{\Delta; \Gamma \vdash t_1\, t_2 \Rightarrow [t_2/x]B; \sigma_2 + s * \sigma_4; \sigma_3 + r * \sigma_4} \Rightarrow \lambda_e$$

The rule can be read by starting at the input of the conclusion (left of $\vdash$), then reading top down through each premise, to calculate the output grades in the rule's conclusion. Any concrete value or already-bound variable appearing in the output grades of a premise can be read as causing an equality check in the type checker. The last premise checks that the output subject-type grade $\sigma_{13}$ from the first premise matches $\sigma_1 + \sigma_3$ (which were calculated by later premises).

In contrast, function introduction is a *check* rule:

$$\frac{\Delta; \Gamma \vdash A \Rightarrow \mathsf{Type}_l; \sigma_1; \mathbf{0} \qquad \Delta, \sigma_1; \Gamma, x : A \vdash t \Leftarrow B; \sigma_2, s; \sigma_3, r}{\Delta; \Gamma \vdash \lambda x.t \Leftarrow (x :_{(s,r)} A) \to B; \sigma_2; \sigma_1 + \sigma_3} \Leftarrow \lambda_i$$

Thus, dependent functions can be checked against type $(x :_{(s,r)} A) \to B$ given input $\Delta; \Gamma$ by first inferring the type of $A$ and checking that its output subject-type grade comprises all zeros $\mathbf{0}$. Then the body of the function $t$ is checked against $B$ under the context $\Delta, \sigma_1; \Gamma, x : A$ producing grade vectors $\sigma_2, s'$ and $\sigma_1, r'$ where it is checked that $s = s'$ and $r = r'$ (described implicitly in the rule), i.e., the calculated grades match those of the binder.

The implementation anticipates some further work for GRTT: the potential for grades which are first-class terms, for which we anticipate complex equations on grades. For grade equality, **Gerty** has two modes: one which normalises terms and then compares for syntactic equality, and the other which discharges constraints via an off-the-shelf SMT solver (we use Z3 [17]). We discuss briefly some performance implications in the next section.

*Using Grades to Optimise Type Checking* Abel posited that a dependent theory with quantitative resource tracking at the type level could leverage linearity-like optimisations in type checking [2]. Our implementation provides a research vehicle for exploring this idea; we consider one possible optimisation here.

Key to dependent type checking is the substitution of terms into types in elimination forms (i.e., application, tensor elimination). However, in a quantitative semiring setting, if a variable has 0 subject-type grade, then we know it is irrelevant to type formation (it is not semantically depended upon, i.e., during normalisation). Subsequently, substitutions into a 0-graded variable can be elided (or allocations to a closure environment can be avoided). We implemented this optimisation in **Gerty** when inferring the type of an application for $t_1\, t_2$ (rule $\Rightarrow \lambda_e$ above), where the type of $t_1$ is inferred as $(x :_{(s,0)} A) \to B$. For a quantitative semiring we know that $x$ irrelevant in $B$, thus we need not perform the substitution $[t_2/x]B$ when type checking the application.

We evaluate this on simple **Gerty** programs of an $n$-ary "fanout" combinator implemented via an $n$-ary application combinator, e.g., for arity 3:

```
app3 : (a : (0, 6) Type 0) -> (b : (0, 2) Type 0)
-> (x0 : (1, 0) a) -> (x1 : (1, 0) a) -> (x2 : (1, 0) a)
-> (f:(1, 0) ((y0:(1,0) a) -> (y1:(1,0) a) -> (y2:(1,0) a) -> b)) -> b
app3 = \a -> \b -> \x0 -> \x1 -> \x2 -> \f -> f x0 x1 x2

fan3 : (a : (0, 4) Type 0) -> (b : (0, 2) Type 0)
-> (f : (1,0) ((z0 : (1,0) a) -> (z1 : (1,0) a) -> (z2 : (1,0) a) -> b))
-> (x : (3, 0) a) -> b
fan3 = \a -> \b -> \f -> \x -> app3 a b x x x f
```

Note that `fan3` uses its parameter `x` three times (hence the grade 3) which then incurs substitutions into the type of `app3` during type checking, but each such substitution is redundant since the type does not depend on these parameters, as reflected by the 0 subject-type grades.

To evaluate the optimisation and SMT solving vs. normalisation-based equality, we ran **Gerty** on the fan out program for arities from 3 to 8, with and without the optimisation and under the two equality approaches.

| | Normalisation | | | SMT | | |
|---|---|---|---|---|---|---|
| $n$ | Base $ms$ | Optimised $ms$ | Speedup | Base $ms$ | Optimised $ms$ | Speedup |
| 3 | 45.71 (1.72) | 44.08 (1.28) | 1.04 | 77.12 (2.65) | 76.91 (2.36) | 1.00 |
| 4 | 108.75 (4.09) | 89.73 (4.73) | 1.21 | 136.18 (5.23) | 162.95 (3.62) | 0.84 |
| 5 | 190.57 (8.31) | 191.25 (8.13) | 1.00 | 279.49 (15.73) | 289.73 (23.30) | 0.96 |
| 6 | 552.11 (29.00) | 445.26 (23.50) | 1.24 | 680.11 (16.28) | 557.08 (13.87) | 1.22 |
| 7 | 1821.49 (49.44) | 1348.85 (26.37) | 1.35 | 1797.09 (43.53) | 1368.45 (20.16) | 1.31 |
| 8 | 6059.30 (132.01) | 4403.10 (86.57) | 1.38 | 5913.06 (118.83) | 4396.90 (59.82) | 1.34 |

**Table 1.** Performance analysis of grade-based optimisations to type checking. Times in milliseconds to 2 d.p. with the standard error given in brackets. Measurements are the mean of 10 trials (run on a 2.7 Ghz Intel Core, 8Gb of RAM, Z3 4.8.8).

Table 1 gives the results. For grade equality by normalisation, the optimisation has a positive effect on speedup, getting increasingly significant (up to 38%) as the overall cost increases. For SMT-based grade equality, the optimisation causes some slow down for arity 4 and 5 (and just breaking even for arity 3). This is because working out whether the optimisation can be applied requires checking whether grades are equal to 0, which incurs extra SMT solver calls. Eventually, this cost is outweighed by the time saved by reducing substitutions. Since the grades here are all relatively simple, it is usually more efficient for the type checker to normalise and compare terms rather than compiling to SMT and starting up the external solver, as seen by longer times for the SMT approach.

The baseline performance here is poor (the implementation is not highly optimised) partly due to the overhead of computing type formation judgments often to accurately account for grading. However, such checks are often recomputed and could be optimised away by memoisation. Nevertheless this experiment gives the evidence that grades can indeed be used to optimise type checking. A thorough investigation of grade-directed optimisations is future work.

## 6 Discussion

*Grading, Coeffects, and Quantitative Types* The notion of *coeffects*, describing how a program depends on its context, arose in the literature from two directions: as a dualisation of effect types [48,49] and a generalisation of Bounded Linear Logic to general resource semirings [25,10]. Coeffect systems can capture reuse bounds, information flow security [23], hardware scheduling constraints [25], and sensitivity for differential privacy [16,22]. A coeffect-style approach also enables linear types to be retrofitted to Haskell [8]. A common thread is the annotation of variables in the context with usage information, drawn from a semiring. Our approach generalises this idea to capture type, context, and computational usage.

McBride [43] reconciles linear and dependent types, allowing types to depend on linear values, refined by Atkey [6] as Quantitative Type Theory. QTT employs coeffect-style annotation of each assumption in a context with an element of a resource accounting algebra, with judgments of the form:

$$x_1 \overset{\rho_1}{:} A_1, \ldots, x_n \overset{\rho_n}{:} A_n \vdash M \overset{\rho}{:} B$$

where $\rho_i, \rho$ are elements of a semiring, and $\rho = 0$ or $\rho = 1$, respectively denoting a term which can be used in type formation (erased at runtime) or at runtime. Dependent function arrows are of the form $(x \overset{\rho}{:} A) \to B$, where $\rho$ is a semiring element that denotes the computational usage of the parameter.

Variables used for type formation but not computation are annotated by 0. Subsequently, type formation rules are all of the form $0\Gamma \vdash T$, meaning every variable assumption has a 0 annotation. GRTT is similar to QTT, but differs in its more extensive grading to track usage in types, rather than blanketing all type usage with 0. In Atkey's formulation, a term can be promoted to a type if its result and dependency quantities are all 0. A set of rules provide formation of computational type terms, but these are also graded at 0. Subsequently, it is not possible to construct an inhabitant of Type that can be used at runtime. We avoid this shortcoming allowing matching on types. For example, a computation $t$ that inspects a type variable $a$ would be typed as: $(\Delta, \mathbf{0}, \Delta' \mid \sigma_1, 1, \sigma_1' \mid \sigma_2, r, \sigma_2') \odot \Gamma, a : \mathsf{Type}, \Gamma' \vdash t : B$ denoting 1 computational use and $r$ type uses in $B$.

At first glance, it seems QTT could be encoded into GRTT taking the semiring $\mathcal{R}$ of QTT and parameterising GRTT by the semiring $\mathcal{R} \cup \{\hat{0}\}$ where $\hat{0}$ denotes arbitrary usage in type formation. However, there is impedance between the two systems as QTT always annotates type use with 0. It is not clear how to make this happen in GRTT whilst still having non-0 tracking at the computational level, since we use one semiring for both. Exploring an encoding is future work.

Choudhury et al. [13] give a system closely related (but arguably simpler) to QTT called GRAD. One key difference is that rather than annotating type usage with 0, grades are simply ignored in types. This makes for a surprisingly flexible system. In addition, they show that irrelevance is captured by the 0 grade using a heap-based semantics (a result leveraged in Section 3). GRAD however does not have the power of type-grades presented here.

*Dependent Types and Modalities* Dal Lago and Gaboardi extend PCF with linear and lightweight dependent types [15] (then adapted for differential privacy analysis [22]). They add a natural number type indexed by upper and lower bound terms which index a modality. Combined with linear arrows of the form $[a < I].\sigma \multimap \tau$ these describe functions using the parameter at most $I$ times (where the modality acts as a binder for index variable $a$ which denotes instantiations). Their system is leveraged to give fine-grained cost analyses in the context of Implicit Computational Complexity. Whilst a powerful system, their approach is restricted in terms of dependency, where only a specialised type can depend on specialised natural-number indexed terms (which are non-linear).

Gratzer et al. define a dependently-typed language with a Fitch-style modality [30]. It seems that such an approach could also be generalised to a graded modality, although we have used the natural-deduction style for our graded modality rather than the Fitch-style.

As discussed in Section 1, our approach closely resembles Abel's *resourceful dependent types* [2]. Our work expands on the idea, including tensors and the graded modalities. We considerably developed the associated metatheory, provide an implementation, and study applications.

*Further Work* One expressive extension is to capture analyses which have an ordering, e.g., grading by a *pre-ordered* semiring, allowing a notion of *approximation*. This would enable analyses such as bounded reuse from Bounded Linear Logic [28], intervals with least- and upper-bounds on use [46], and top-completed semirings, with an $\infty$-element denoting arbitrary usage as a fall-back. We have made progress into exploring the interaction between approximation and dependent types, and the remainder of this is left as future work.

A powerful extension of GRTT for future work is to allow grades to be first-class terms. Typing rules in GRTT involving grades could be adapted to internalise the elements as first-class terms. We could then, e.g., define the map function over sized vectors, which requires that the parameter function is used exactly the same number of times as the length of the vector:

$$\begin{aligned} map : (n :_{(0,5)} \mathsf{nat}) \to (a :_{(0,n+1)} \mathsf{Type}) \to (b :_{(0,n+1)} \mathsf{Type}) \to \\ (f :_{(n,0)} (x :_{(1,0)} a) \to b) \to (xs :_{(1,0)} \mathsf{Vec}\, n\, a) \to \mathsf{Vec}\, n\, b \end{aligned}$$

This type provides strong guarantees: the only well-typed implementations do the correct thing, up to permutations of the result vector. Without the grading, an implementation could apply $f$ fewer than $n$ times, replicating some of the transformed elements; here we know that $f$ must be applied exactly $n$-times.

A further appealing possibility for GRTT is to allow the semiring to be defined internally, rather than as a meta-level parameter, leveraging dependent types for proofs of key properties. An implementation could specify what is required for a semiring instance, e.g., a record type capturing the operations and properties of a semiring. The rules of GRTT could then be extended, similarly to the extension to first-class grades, with the provision of the semiring(s) coming from GRTT terms. Thus, anywhere with a grading premise $(\Delta \mid \sigma_1 \mid \sigma_2) \odot \Gamma \vdash r : \mathcal{R}$ would also require a premise $(\Delta \mid \sigma_2 \mid \mathbf{0}) \odot \Gamma \vdash \mathcal{R} : \mathsf{Semiring}$. This opens up the ability for programmers and library developers to provide custom modes of resource tracking with their libraries, allowing domain-specific program verification.

*Conclusions* The paradigm of 'grading' exposes the inherent structure of a type theory, proof theory, or semantics by matching the underlying structure with some algebraic structure augmenting the types. This idea has been employed for reasoning about side effects via graded monads [35], and reasoning about data flow as discussed here by semiring grading. Richer algebras could be employed to capture other aspects, such as *ordered logics* in which the exchange rule can be controlled via grading (existing work has done this via modalities [34]).

We developed the core of grading in the context of dependent-types, treating types and terms equally (as one comes to expect in dependent-type theories). The tracking of data flow in types appears complex since we must account for how variables are used to form types in both the context and in the subject type, making sure not to repeat context formation use. The result however is a powerful system for studying dependencies in type theories, as shown by our ability to study different theories just be specialising grades. Whilst not yet a fully fledged implementation, **Gerty** is a useful test bed for further exploration.

*Acknowledgments* Orchard is supported by EPSRC grant EP/T013516/1.
